# Temperature Effects Explain Continental Scale Distribution of Cyanobacterial Toxins

**DOI:** 10.3390/toxins10040156

**Published:** 2018-04-13

**Authors:** Evanthia Mantzouki, Miquel Lürling, Jutta Fastner, Lisette de Senerpont Domis, Elżbieta Wilk-Woźniak, Judita Koreivienė, Laura Seelen, Sven Teurlincx, Yvon Verstijnen, Wojciech Krztoń, Edward Walusiak, Jūratė Karosienė, Jūratė Kasperovičienė, Ksenija Savadova, Irma Vitonytė, Carmen Cillero-Castro, Agnieszka Budzyńska, Ryszard Goldyn, Anna Kozak, Joanna Rosińska, Elżbieta Szeląg-Wasielewska, Piotr Domek, Natalia Jakubowska-Krepska, Kinga Kwasizur, Beata Messyasz, Aleksandra Pełechata, Mariusz Pełechaty, Mikolaj Kokocinski, Ana García-Murcia, Monserrat Real, Elvira Romans, Jordi Noguero-Ribes, David Parreño Duque, Elísabeth Fernández-Morán, Nusret Karakaya, Kerstin Häggqvist, Nilsun Demir, Meryem Beklioğlu, Nur Filiz, Eti E. Levi, Uğur Iskin, Gizem Bezirci, Ülkü Nihan Tavşanoğlu, Koray Özhan, Spyros Gkelis, Manthos Panou, Özden Fakioglu, Christos Avagianos, Triantafyllos Kaloudis, Kemal Çelik, Mete Yilmaz, Rafael Marcé, Nuria Catalán, Andrea G. Bravo, Moritz Buck, William Colom-Montero, Kristiina Mustonen, Don Pierson, Yang Yang, Pedro M. Raposeiro, Vítor Gonçalves, Maria G. Antoniou, Nikoletta Tsiarta, Valerie McCarthy, Victor C. Perello, Tõnu Feldmann, Alo Laas, Kristel Panksep, Lea Tuvikene, Ilona Gagala, Joana Mankiewicz-Boczek, Meral Apaydın Yağcı, Şakir Çınar, Kadir Çapkın, Abdulkadir Yağcı, Mehmet Cesur, Fuat Bilgin, Cafer Bulut, Rahmi Uysal, Ulrike Obertegger, Adriano Boscaini, Giovanna Flaim, Nico Salmaso, Leonardo Cerasino, Jessica Richardson, Petra M. Visser, Jolanda M. H. Verspagen, Tünay Karan, Elif Neyran Soylu, Faruk Maraşlıoğlu, Agnieszka Napiórkowska-Krzebietke, Agnieszka Ochocka, Agnieszka Pasztaleniec, Ana M. Antão-Geraldes, Vitor Vasconcelos, João Morais, Micaela Vale, Latife Köker, Reyhan Akçaalan, Meriç Albay, Dubravka Špoljarić Maronić, Filip Stević, Tanja Žuna Pfeiffer, Jeremy Fonvielle, Dietmar Straile, Karl-Otto Rothhaupt, Lars-Anders Hansson, Pablo Urrutia-Cordero, Luděk Bláha, Rodan Geriš, Markéta Fránková, Mehmet Ali Turan Koçer, Mehmet Tahir Alp, Spela Remec-Rekar, Tina Elersek, Theodoros Triantis, Sevasti-Kiriaki Zervou, Anastasia Hiskia, Sigrid Haande, Birger Skjelbred, Beata Madrecka, Hana Nemova, Iveta Drastichova, Lucia Chomova, Christine Edwards, Tuğba Ongun Sevindik, Hatice Tunca, Burçin Önem, Boris Aleksovski, Svetislav Krstić, Itana Bokan Vucelić, Lidia Nawrocka, Pauliina Salmi, Danielle Machado-Vieira, Alinne Gurjão de Oliveira, Jordi Delgado-Martín, David García, Jose Luís Cereijo, Joan Gomà, Mari Carmen Trapote, Teresa Vegas-Vilarrúbia, Biel Obrador, Magdalena Grabowska, Maciej Karpowicz, Damian Chmura, Bárbara Úbeda, José Ángel Gálvez, Arda Özen, Kirsten Seestern Christoffersen, Trine Perlt Warming, Justyna Kobos, Hanna Mazur-Marzec, Carmen Pérez-Martínez, Eloísa Ramos-Rodríguez, Lauri Arvola, Pablo Alcaraz-Párraga, Magdalena Toporowska, Barbara Pawlik-Skowronska, Michał Niedźwiecki, Wojciech Pęczuła, Manel Leira, Armand Hernández, Enrique Moreno-Ostos, José María Blanco, Valeriano Rodríguez, Jorge Juan Montes-Pérez, Roberto L. Palomino, Estela Rodríguez-Pérez, Rafael Carballeira, Antonio Camacho, Antonio Picazo, Carlos Rochera, Anna C. Santamans, Carmen Ferriol, Susana Romo, Juan Miguel Soria, Julita Dunalska, Justyna Sieńska, Daniel Szymański, Marek Kruk, Iwona Kostrzewska-Szlakowska, Iwona Jasser, Petar Žutinić, Marija Gligora Udovič, Anđelka Plenković-Moraj, Magdalena Frąk, Agnieszka Bańkowska-Sobczak, Michał Wasilewicz, Korhan Özkan, Valentini Maliaka, Kersti Kangro, Hans-Peter Grossart, Hans W. Paerl, Cayelan C. Carey, Bas W. Ibelings

**Affiliations:** 1 Department F.-A. Forel for Environmental and Aquatic Sciences, University of Geneva, 1205 Geneva, Switzerland; Bastiaan.Ibelings@unige.ch; 2 Department of Environmental Sciences, Wageningen University & Research, 6700 Wageningen, The Netherlands; miquel.lurling@wur.nl (M.L.); l.desenerpontdomis@nioo.knaw.nl (L.d.S.D.); l.seelen@nioo.knaw.nl (L.S.); yvonverstijnen@hotmail.com (Y.V.); valentini.maliaka@gmail.com (V.M.); 3 Department of Aquatic Ecology, Netherlands Institute of Ecology (NIOO-KNAW), 6700 Wageningen, The Netherlands; s.teurlincx@nioo.knaw.nl; 4 German Environment Agency, Unit Drinking Water Resources and Water Treatment, Corrensplatz 1, 14195 Berlin, Germany; jutta.fastner@uba.de; 5 Institute of Nature Conservation, Polish Academy of Sciences, 31-120 Krakow, Poland; wilk@iop.krakow.pl (E.W.-W.); krzton@iop.krakow.pl (W.K.); walusiak@iop.krakow.pl (E.W.); 6 Institute of Botany, Nature Research Centre, Vilnius 08412, Lithuania; judita.koreiviene@gmail.com (J.K.); jurate.karosiene@botanika.lt (J.K.); jurate.kasperoviciene@botanika.lt (J.K.); ksenija.savadova@gamtostyrimai.lt (K.S.); irma.vitonyte@gmail.com (I.V.); 7 R&D Department Environmental Engineering, 3edata, 27004 Lugo, Spain; carmen.cillero@3edata.es; 8 Department of Water Protection, Adam Mickiewicz University, 61614 Poznan, Poland; ag.budzynska@gmail.com (A.B.); rgold@amu.edu.pl (R.G.); akozak@amu.edu.pl (A.K.); rosinska.asia@gmail.com (J.R.); eszelag@amu.edu.pl (E.S.-W.); domekp@amu.edu.pl (P.D.); jakubowskan@gmail.com (N.J.-K.); 9 Department of Hydrobiology, Adam Mickiewicz University, 61614 Poznan, Poland; kingakwasizur@wp.pl (K.K.); messyasz@amu.edu.pl (B.M.); ola.p@amu.edu.pl (A.P.); marpel@amu.edu.pl (M.P.); kok@amu.edu.pl (M.K.); 10 Department of Limnology and Water Quality, AECOM U.R.S, 08036 Barcelona, Spain; ana.garcia@aecom.com (A.G.-M.); montserrat.real@aecom.com (M.R.); elvira.romans@aecom.com (E.R.); jordi.noguero@aecom.com (J.N.-R.); parra1404@hotmail.com (D.P.D.); elifdzmo@gmail.com (E.F.-M.); 11 Department of Environmental Engineering, Abant Izzet Baysal University, 14280 Bolu, Turkey; karakaya_n@ibu.edu.tr; 12 Department of Science and Engineering, Åbo Akademi University, 20520 Åbo, Finland; kerstin.haggqvist@gmail.com; 13 Department of Fisheries and Aquaculture, Ankara University, 6100 Ankara, Turkey; nilsundemir2@gmail.com; 14 Department of biology, Middle East Technical University, 6800 Ankara, Turkey; meryem@metu.edu.tr (M.B.); nrflzster@gmail.com (N.F.); eti@thelevis.com (E.E.L.); iskin.ugur@gmail.com (U.I.); gizbezirci@gmail.com (G.B.); unyazgan@gmail.com (Ü.N.T.); 15 Institute of Marine Sciences, Department of Oceanography, Middle East Technical University, 06800 Ankara, Turkey; koray@ims.metu.edu.tr; 16 Department of Botany, Aristotle University of Thessaloniki, 54124 Thessaloniki, Greece; sgkelis@bio.auth.gr (S.G.); mattpano@bio.auth.gr (M.P.); 17 Department of Basic Science, Ataturk University, 25240 Erzurum, Turkey; ozden.fakioglu@atauni.edu.tr; 18 Water Quality Department, Athens Water Supply and Sewerage Company, 11146 Athens, Greece; c.avagianos@icloud.com (C.A.); t_kaloudis@ath.forthnet.gr (T.K.); 19 Department of Biology, Balikesir University, 10145 Balikesir, Turkey; kcelik@balikesir.edu.tr; 20 Department of Bioengineering, Bursa Technical University, 16310 Bursa, Turkey; mete.yilmaz@btu.edu.tr; 21 Catalan Institute for Water Research (ICRA), 17003 Girona, Spain; rmarce@icra.cat (R.M.); ncatalangarcia@gmail.com (N.C.); 22 Department of Ecology and Genetics, Limnology, Uppsala University, 75236 Uppsala, Sweden; jandriugarcia@gmail.com (A.G.B.); moritz.buck@ebc.uu.se (M.B.); pablo.urrutia_cordero@biol.lu.se (P.U.-C.); 23 Department of Ecology and Genetics, Erken Laboratory, Uppsala University, 76173 Norrtalje, Sweden; william.colom@ebc.uu.se (W.C.-M.); kristiina.mustonen@ebc.uu.se (K.M.); don.pierson@ebc.uu.se (D.P.); yang.yang@hotmail.se (Y.Y.); 24 Research Center in Biodiversity and Genetic Resources (CIBIO-Azores), InBIO Associated Laboratory, Faculty of Sciences and Technology, University of the Azores, 9501-801 Ponta Delgada, Portugal; pedro.mv.raposeiro@uac.pt (P.M.R.); vitor.mc.goncalves@uac.pt (V.G.); 25 Department of Environmental Science and Technology, Cyprus University of Technology, 3036 Lemesos, Cyprus; maria.antoniou@cut.ac.cy (M.G.A.); tsiarta.nikoletta@gmail.com (N.T.); 26 Centre for Freshwater and Environmental Studies, Dundalk Institute of Technology, A91 K584 Dundalk, Ireland; valerie.mccarthy@dkit.ie (V.M.); victorc.perello@dkit.ie (V.C.P.); 27 Institute of Agricultural and Environmental Sciences, Estonian University of Life Sciences, 51014 Tartu, Estonia; tonu.feldmann@emu.ee (T.F.); alo.laas@emu.ee (A.L.); kristel.panksep@emu.ee (K.P.); lea.tuvikene@emu.ee (L.T.); kiti@ut.ee (K.K.); 28 European Regional Centre for Ecohydrology of the Polish Academy of Sciences, 90364 Lodz, Poland; ilonagagala@erce.unesco.lodz.pl (I.G.); j.mankiewicz@erce.unesco.lodz.pl (J.M.-B.); 29 Republic of Turkey Ministry of Food Agriculture, Fisheries Research Institute, 32500 Eğirdir, Isparta, Turkey; meralyagci@gmail.com (M.A.Y.); sakircinar@gmail.com (Ş.Ç.); kadircapkin32@hotmail.com (K.Ç.); a.k.yagci58@gmail.com (A.Y.); mehmetcesur3232@gmail.com (M.C.); fuatbilgin@gmail.com (F.B.); caferbulut@gmail.com (C.B.); uysalrahmi@hotmail.com (R.U.); 30 Department of Sustainable Ecosystems and Bioresources, Fondazione Edmund Mach, 38010 San Michele all’Adige, Italy; ulrike.obertegger@fmach.it (U.O.); adriano.boscaini@fmach.it (A.B.); giovanna.flaim@fmach.it (G.F.); nico.salmaso@fmach.it (N.S.); leonardo.cerasino@fmach.it (L.C.); 31 Department of Biological and Environmental Sciences, University of Stirling, Stirling FK9 4LA, UK; jerich15@ceh.ac.uk; 32 Department of Freshwater and Marine Ecology, University of Amsterdam, 1090 GE Amsterdam, The Netherlands; p.m.visser@uva.nl (P.M.V.); j.m.h.verspagen@uva.nl (J.M.H.V.); 33 Department of Molecular Biology and Genetics, Gaziosmanpasa University, 60250 Merkez, Turkey; biyo_tunay@hotmail.com; 34 Department of Biology, Giresun University, 28100 Giresun, Turkey; elif.neyran.soylu@giresun.edu.tr; 35 Department of Biology, Hitit University, 19040 Corum, Turkey; farukmaraslioglu@hitit.edu.tr; 36 Department of Icthyology, Hydrobiology and Aquatic Ecology, Inland Fisheries Institute, 10719 Olsztyn, Poland; a.napiorkowska-krzebietke@infish.com.pl; 37 Department of Freshwater Protection, Institute of Environmental Protection- National Research Institute, 01-692 Warsaw, Poland; a.ochocka@ios.edu.pl (A.O.); paszta@ios.edu.pl (A.P.); 38 Centro de Investigação da Montanha, Instituto Politécnico de Bragança, Campus de Santa Apolónia, 5300-253 Bragança, Portugal; geraldes@ipb.pt; 39 Interdisciplinary Centre of Marine and Environmental Research (CIIMAR) and University of Porto, 4450-208 Matosinhos, Portugal; vmvascon@fc.up.pt (V.V.); joaopmorais@gmail.com (J.M.); mica.ciimar@gmail.com (M.V.); 40 Department of Freshwater Resource and Management, Faculty of Aquatic Sciences, Istanbul University, 34134 Istanbul, Turkey; latifekoker@gmail.com (L.K.); rakcaalan@gmail.com (R.A.); albay.hermano@gmail.com (M.A.); 41 Department of Biology, Josip Juraj Strossmayer University of Osijek, 31000 Osijek, Croatia; dspoljaric@biologija.unios.hr (D.Š.M.); fstevic@biologija.unios.hr (F.S.); tzuna@biologija.unios.hr (T.Ž.P.); 42 Department of Experimental Limnology, Leibniz Institute of Freshwater Ecology and Inland Fisheries, 16775 Stechlin, Germany; fonvielle@igb-berlin.de (J.F.); hgrossart@igb-berlin.de (H.-P.G.); 43 Department of Biology, Limnological Institute, University of Konstanz, 78464 Konstanz, Germany; dietmar.straile@uni-konstanz.de (D.S.); karl.rothhaupt@uni-konstanz.de (K.-O.R.); 44 Department of Biology, Lund University, 22362 Lund, Sweden; lars-anders.hansson@biol.lu.se; 45 RECETOX, Faculty of Science, Masaryk University, 62500 Brno, Czech Republic; blaha@recetox.muni.cz; 46 Department of Hydrobiology, Morava Board Authority, 60200 Brno, Czech Republic; geris@pmo.cz; 47 Laboratory of Paleoecology, Institute of Botany, The Czech Academy of Sciences, 60200 Brno, Czech Republic; marketka.kozakova@seznam.cz; 48 Department of Environment and Resource Management, Mediterranean Fisheries Research Production and Training Institute, 7090 Antalya, Turkey; matkocer@hotmail.com; 49 Faculty of Aquaculture, Mersin University, 33160 Mersin, Turkey; talp@mersin.edu.tr; 50 Department of Water Quality, Slovenian Environmental Agency, 1000 Ljubljana, Slovenia; spela.remec-rekar@gov.si; 51 Department of Genetic Toxicology and Cancer Biology, National Institute of Biology, 1000 Ljubljana, Slovenia; tina.elersek@nib.si; 52 Institute of Nanoscience and Nanotechnology, National Center for Scientific Research «DEMOKRITOS», 15341 Attiki, Greece; t.triantis@inn.demokritos.gr (T.T.); s.zervou@inn.demokritos.gr (S.-K.Z.); a.hiskia@inn.demokritos.gr (A.H.); 53 Department of Freshwater Ecology, Norwegian Institute for Water Research, 0349 Oslo, Norway; sigrid.haande@niva.no (S.H.); birger.skjelbred@niva.no (B.S.); 54 Institute of Environmental Engineering, Poznan University of Technology, 60965 Poznan, Poland; beata.madrecka@put.poznan.pl; 55 National Reference Center for Hydrobiology, Public Health Authority of the Slovak Republic, 82645 Bratislava, Slovakia; hana.nemova@uvzsr.sk (H.N.); iveta.drastichova@uvzsr.sk (I.D.); lucia.chomova@uvzsr.sk (L.C.); 56 School of Pharmacy and Life Sciences, Robert Gordon University, Aberdeen AB10 7GJ, UK; c.edwards@rgu.ac.uk; 57 Department of Biology, Sakarya University, 54187 Sakarya, Turkey; tsevindik@sakarya.edu.tr (T.O.S.); htunca@sakarya.edu.tr (H.T.); burcin.onem1@ogr.sakarya.edu.tr (B.Ö.); 58 Faculty of Natural Sciences and Mathematics, SS Cyril and Methodius University, 1000 Skopje, Macedonia; borisaleksovski@pmf.ukim.mk (B.A.); skrstic@pmf.ukim.mk (S.K.); 59 Department for Ecotoxicology, Teaching Institute of Public Health of Primorje-Gorski Kotar County, 51000 Rijeka, Croatia; itana.bokan@zzjzpgz.hr; 60 Institute of Technology, The State University of Applied Sciences, 82300 Elblag, Poland; nawrocka.lidia@op.pl; 61 Department of Biological and Environmental Science, University of Jyväskylä, 40014 Jyväskylä, Finland; pauliina.u.m.salmi@jyu.fi; 62 Departamento de Sistemática e Ecologia, Universidade Federal da Paraíba, 58059-970 Paraíba, Brasil; daniellemachadovieira86@gmail.com (D.M.-V.); alinnegurjao@gmail.com (A.G.d.O.); 63 Department of Civil Engineering, University of A Coruña, 15192 A Coruña, Spain; jdelgado@udc.es (J.D.-M.); david.morrondo@udc.es (D.G.); jose.cereijo@udc.es (J.L.C.); 64 Department of Evolutionary Biology, Ecology, and Environmental Sciences, University of Barcelona, 08028 Barcelona, Spain; jgoma@ub.edu (J.G.); mctrapote84@gmail.com (M.C.T.); tvegas@ub.edu (T.V.-V.); obrador@ub.edu (B.O.); 65 Department of Hydrobiology, University of Bialystok, 15245 Bialystok, Poland; magra@uwb.edu.pl (M.G.); m.karpowicz@uwb.edu.pl (M.K.); 66 Institute of Environmental Protection and Engineering, University of Bielsko-Biala, 43309 Bielsko-Biala, Poland; dachmura@gmail.com; 67 Department of Biology, University of Cádiz, 11510 Puerto Real, Cádiz, Spain; barbara.ubeda@uca.es (B.Ú.); joseangel.galvez@uca.es (J.Á.G.); 68 Department of Forest Engineering, University of Cankiri Karatekin, 18200 Cankiri, Turkey; ardaozen@gmail.com; 69 Freshwater Biological Laboratory, Department of Biology, University of Copenhagen, 2100 Copenhagen, Denmark; kchristoffersen@bio.ku.dk (K.S.C.); twperlt@bio.ku.dk (T.P.W.); 70 Department of Marine Biotechnology, University of Gdansk, 81378 Gdynia, Poland; justyna.kobos@ug.edu.pl (J.K.); biohm@ug.edu.pl (H.M.-M.); 71 Department of Ecology, University of Granada, 18071 Granada, Spain; cperezm@ugr.es (C.P.-M.); eloisa@ugr.es (E.R.-R.); 72 Lammi Biological Station, University of Helsinki, 16900 Lammi, Finland; lauri.arvola@helsinki.fi; 73 Department of Animal Biology, Plant Biology and Ecology, University of Jaen, 23701 Jaen, Spain; pabloalcarazparraga@gmail.com; 74 Department of Hydrobiology and Protection of Ecosystems, University of Life Sciences in Lublin, 20262 Lublin, Poland; magda.wis@interia.pl (M.T.); barbara.pawlik@up.lublin.pl (B.P.-S.); michal.niedzwiecki@up.lublin.pl (M.N.); wojciech.peczula@up.lublin.pl (W.P.); 75 Instituto Dom Luiz, University of Lisbon, 1749016 Lisbon, Portugal; mleira@fc.ul.pt; 76 Institute of Earth Sciences Jaume Almera, ICTJA, CSIC, 08028 Barcelona, Spain; ahernandez@ictja.csic.es; 77 Department of Ecology, University of Malaga, 29071 Malaga, Spain; quique@uma.es (E.M.-O.); jmblanco@uma.es (J.M.B.); valeriano@uma.es (V.R.); montesperezjj@gmail.com (J.J.M.-P.); rlpalomino@uma.es (R.L.P.); stelax_rp@hotmail.com (E.R.-P.); 78 Centro de Investigacións Cientificas Avanzadas (CICA), Facultade de Ciencias, Universidade da Coruña, 15071 A Coruña, Spain; r.carballeira@udc.es; 79 Cavanilles Institute of Biodiversity and Evolutionary Biology, University of Valencia, 46980 Paterna Valencia, Spain; antonio.camacho@uv.es (A.C.); antonio.picazo-mozo@uv.es (A.P.); carlos.rochera@uv.es (C.R.); anna.santamans@gmail.com (A.C.S.); carmen.ferriol@uv.es (C.F.); 80 Department of Microbiology and Ecology, University of Valencia, 46100 Burjassot, Spain; susana.romo@uv.es (S.R.); juan.soria@uv.es (J.M.S.); 81 Department of Water Protection Engineering, University of Warmia and Mazury, 10-720 Olsztyn, Poland; julitad@uwm.edu.pl (J.D.); justyna.sienska@uwm.edu.pl (J.S.); daniel.szymanski@uwm.edu.pl (D.S.); 82 Department of Tourism, Recreation and Ecology, University of Warmia and Mazury, 10-720 Olsztyn, Poland; mkruk@uwm.edu.pl; 83 Faculty of Biology, University of Warsaw, 02-096 Warsaw, Poland; iwona.ks@biol.uw.edu.pl; 84 Department of Plant Ecology and Environmental Conservation, Faculty of Biology, University of Warsaw, 02-089 Warsaw, Poland; jasser.iwona@biol.uw.edu.pl; 85 Department of Biology, Faculty of Science, University of Zagreb, 10000 Zagreb, Croatia; petar.zutinic@biol.pmf.hr (P.Ž.); marija.gligora.udovic@biol.pmf.hr (M.G.U.); andjelka.plenkovic-moraj@biol.pmf.hr (A.P.-M.); 86 Department of Environmental Improvement, Faculty of Civil and Environmental Engineering, Warsaw University of Life Sciences—SGGW, 02-787 Warsaw, Poland; magdalena_frak@sggw.pl; 87 Department of Hydraulic Engineering, Faculty of Civil and Environmental Engineering, Warsaw University of Life Sciences—SGGW, 02-787 Warsaw, Poland; agnieszka_bankowska@sggw.pl (A.B.-S.); michal_wasilewicz@sggw.pl (M.W.); 88 Institute of Marine Sciences, Marine Biology and Fisheries, Middle East Technical University, 06800 Ankara, Turkey; okorhan@metu.edu.tr; 89 Society for the Protection of Prespa, 53077 Agios Germanos, Greece; 90 Institute for Water and Wetland Research, Department of Aquatic Ecology and Environmental Biology, Radboud University Nijmegen, 6525 AJ Nijmegen, The Netherlands; 91 Tartu Observatory, Faculty of Science and Technology, University of Tartu, 61602 Tartu, Estonia; 92 Institute of Biochemistry and Biology, Potsdam University, 14469 Potsdam, Germany; 93 Institute of Marine Sciences, University of North Carolina at Chapel Hill, Chapel Hill, NC 28557, USA; hpaerl@email.unc.edu; 94 Department of Biological Sciences, Virginia Tech, Blacksburg, VA 24061, USA; cayelan@vt.edu

**Keywords:** microcystin, anatoxin, cylindrospermopsin, temperature, direct effects, indirect effects, spatial distribution, European Multi Lake Survey

## Abstract

Insight into how environmental change determines the production and distribution of cyanobacterial toxins is necessary for risk assessment. Management guidelines currently focus on hepatotoxins (microcystins). Increasing attention is given to other classes, such as neurotoxins (e.g., anatoxin-a) and cytotoxins (e.g., cylindrospermopsin) due to their potency. Most studies examine the relationship between individual toxin variants and environmental factors, such as nutrients, temperature and light. In summer 2015, we collected samples across Europe to investigate the effect of nutrient and temperature gradients on the variability of toxin production at a continental scale. Direct and indirect effects of temperature were the main drivers of the spatial distribution in the toxins produced by the cyanobacterial community, the toxin concentrations and toxin quota. Generalized linear models showed that a Toxin Diversity Index (TDI) increased with latitude, while it decreased with water stability. Increases in TDI were explained through a significant increase in toxin variants such as MC-YR, anatoxin and cylindrospermopsin, accompanied by a decreasing presence of MC-LR. While global warming continues, the direct and indirect effects of increased lake temperatures will drive changes in the distribution of cyanobacterial toxins in Europe, potentially promoting selection of a few highly toxic species or strains.

## 1. Introduction

As a consequence of human population growth, along with associated agricultural, urban and industrial activities, harmful algal blooms worldwide are on the increase [1]. Eutrophication, one major outcome of anthropogenic activities in the catchments of aquatic ecosystems, is consistently recognized as the main driver of cyanobacterial blooms [2,3]. In addition, damage to ecosystems and loss of natural resources (e.g., in Lake Taihu, China [4] and references within [5]) are also attributed to on-going climatic change [6]. A synergistic interaction between increased nutrients and climate-related changes is predicted [7] based on experimental [8] and field studies [9], potentially further exacerbating the occurrence of cyanobacterial blooms. 

The long history of cyanobacterial adaptation to a wide range of environmental conditions including extremes [10] supports their successful occurrence in a variety of lake ecosystems. These adaptations come in the form of functional traits such as phosphorus storage, buoyancy regulation, nitrogen fixation and the formation of akinetes (resting spores). Extensive research has linked the prevalence of species with specific functional traits to certain sets of environmental conditions [11,12]. For example, *Microcystis aeruginosa* can rapidly float up in the illuminated near surface layers under conditions of enhanced water column stability [13], and through buoyancy regulation gain access to both nutrients at deeper layers and light at the surface [14]. 

Toxin production, by the production of hepatotoxins (e.g., microcystins (MCs) and nodularin (NOD)), cytotoxins (cylindrospermopsins (CYN)) and neurotoxins (e.g., anatoxins (ATX)), is another common trait of a large number of cyanobacterial species. Although numerous studies have elucidated the chemical properties, biosynthesis and genetics of the most well-known toxins [15,16,17], still little is known why toxins are produced and what determines their presence in field populations. There is evidence that production of these secondary metabolites might provide a competitive advantage for example through providing resistance against grazing [18,19,20] or a physiological benefit e.g., in enhancing nutrient uptake or offering protection against oxidative stress (references within [21]). The abundance of toxins during blooms depends on the presence of toxic strains within the cyanobacterial population [22]. Different species have been shown to produce specific toxins or even variants [23,24,25]. However, in toxic strains, the environmental factors controlling expression of the toxin synthetase genes are still a contentious issue [26]. Lack of consistency in experimental findings, along with a lack of standardization in surveying and sampling design in field studies, so far has hindered a coherent understanding of how environmental stressors are linked to cyanobacterial toxin production and toxin quota (toxin concentration per cell or unit algal biomass). 

Various studies have shown contradictory responses of toxin producing taxa to similar environmental parameters. For example, experiments with MC-producing *Planktothrix agardhii* showed that high nitrogen concentrations (one factor in the study to vary among others like phosphorus, temperature, pH, light) were correlated to high MC production in batch cultures [27]. However, in experiments with *Microcystis aeruginosa*, N-limited chemostat experiments triggered an increase in MC content, with smaller and faster growing cells being mostly promoted as a response to favourable growing conditions [28]. Similarly, an experiment with ATX producers showed that temperature dependent optimal growth conditions did not necessarily result in higher toxin concentration [29]. In this study, while *Aphanizomenon* cultures proliferated at 30 °C, *Dolichospermum* (formerly *Anabaena*) cultures suffered at this high temperature [29]. Nevertheless, ATX production was reduced by both tested species [29]. A subsequent study on ATX or MC producing strains of *Dolichospermum* indicated strain-specific responses to temperature and light limited conditions [30]. Consequently, there is no easy way to deduce from environmental conditions which toxins will come to dominate in a bloom, nor whether toxin concentrations will be high.

Microcystins (MCs), as the largest, best described and most diverse group of cyanobacterial toxins [26], have been the focus of management and mitigation guidelines. For drinking water, the World Health Organisation (WHO) has set a provisional guideline value-maintained at the same level in the EU Drinking Water Directive-of 1 µg/L for MC-LR, a value that is accepted in most countries. In recreational waters, however, there is less consistency, even for MC-LR, and national authorities use a variety of risk assessment schemes and criteria to inform management decisions/practice [31]. MC-LR is the best studied MC variant [32,33], yet other variants such as MC-YR [34], -LW and -LF [35] can also be highly toxic and may contribute significantly to the total MCs in a lake. MC-RR, although reported to be ten times less toxic than MC-LR after intraperitoneal injection in mice (LD_50_), is one of the most frequently reported toxin in the lakes along with MC-LR and MC-YR [36] and may be more harmful to aquatic biota than MC-LR [37]. Good toxicity data are lacking for the vast majority of MC variants (presently more than 250 [23]). Good toxicological data on other toxins such as CYN and ATX are necessary to include a full spectrum of cyanotoxins in human and ecosystem risk assessment (reviewed in [36,38]).

Although a fair number of toxin surveys at the national level have been carried out [39], studies have rarely investigated the spatial distribution of different classes of cyanotoxins at larger geographical scales, encompassing lakes of widely different characteristics and environmental diversity. According to studies [40,41], the climate in Europe is already shifting north (e.g., central European countries will experience longer hot summers, similar to presently in Mediterranean countries) and, as such, a study on cyanotoxins over a large latitudinal gradient may offer insights into their future distribution. In the European Multi Lake Survey (EMLS), lakes across the continent were sampled once in a snapshot approach, for physical, chemical and biological parameters, during the summer of 2015. Standardized and commonly practiced field protocols along with centralized laboratory analyses for all parameters other than microscopy were undertaken for all samples, avoiding inconsistencies between data in this large dataset. In addition, we addressed our research questions minimizing confounding effects of seasonality, by sampling all lakes during the locally two warmest weeks of summer based on at least 10-year air temperature records. In this study, we investigated how the distribution of toxin concentration and toxin quota were defined by environmental parameters. We hypothesize that, in an unusually warm summer—like 2015—was in parts of Europe [42]—temperature, either through direct (surface temperature, epilimnetic temperature) or indirect (water stability expressed as maximum buoyancy frequency) effects, strongly influences the distribution of toxin concentrations and toxin quota. Additionally, we hypothesize that, under high temperature stress, the stringent selection of specific well-adapted strains of cyanobacteria reduces toxin diversity, potentially promoting dominance by a few highly toxic variants.

## 2. Results

### 2.1. Toxin Distribution on a Continental Scale

In the subset of the 137 EMLS lakes used in our analysis, all seven toxins analysed were detected in samples from only three lakes, which shows that it is possible but rare to have such a diverse number of toxins present in one lake at one moment in time. The presence of four, five or six toxins was found in 34, 26 and 25 lakes, respectively. Finally, 18 lakes had two toxins and 13 lakes only one toxin. 

MC variants were found in 93% of the 137 EMLS lakes (Table 1). MC-YR was the most common of the five MCs (in 82% of the subset). Although the variant MC-dmRR was the rarest variant encountered, it had the highest concentrations compared to any other toxin variant (14.89 µg/L in Polish Lake Syczyńskie). The MC variants MC-LW, MC-LF and MC-LY, and the toxin nodularin (NOD) were also analysed, but they were not included in the analysis as they were too scarce (see materials and methods). The MC-LF was present in two Spanish reservoirs (Abegondo and As Forcadas), while MC-LW and MC-LY were not found in any of the EMLS lakes. Nodularin was found only in two Spanish reservoirs (As Forcadas and Valdecanas) in concentrations close to the limit of quantification (0.007 µg/L). 

**Figure 1:**
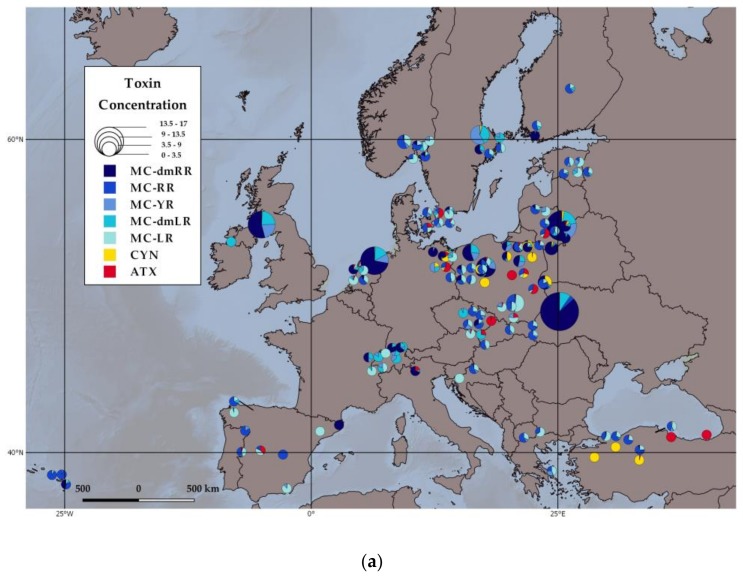

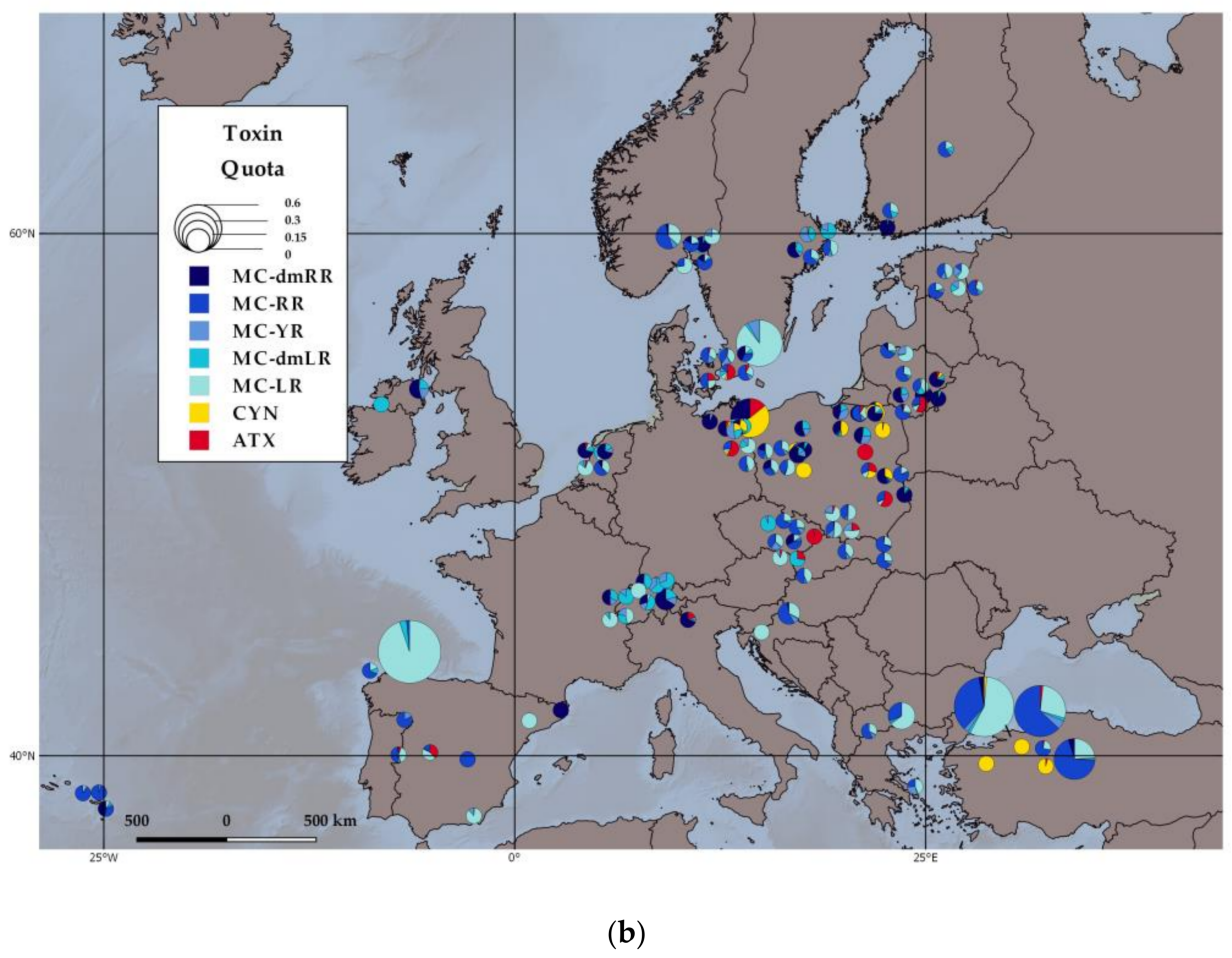
Percentages of (**a**) toxin concentrations (μg/L) and (**b**) toxin quota (μg toxin/μg chlorophyll-a) of each toxin, of the 137 EMLS lakes used in the analyses. Blue shades correspond to the five microcystin variants (MC-YR; MC-dmLR; MC-LR; MC-RR; MC-dmRR), yellow to cylindrospermospin (CYN) and red to anatoxin (ATX). The radius of the pie charts corresponds to (**a**) the total toxin concentrations and (**b**) to the total toxin quota.

CYN was detected in 39% of the 137 EMLS lakes (Table 1). One German Lake (Grosser Dabelowsee), three Polish Lakes (Bnińskie, Lusowskie and Probarskie), and two Turkish lakes (Caycoren and Mollakoy) (Figure 1a) had solely CYN, in relatively low concentrations (<0.05 µg/L, supplemental material). ATX was found in 39% of the EMLS lakes (Table 1), out of which only one Polish Lake (Dziekanowskie) (Figure 1a) produced the specific toxin exclusively, albeit at very low concentrations 0.002 µg/L. 

### 2.2. Multivariate Multiple Regression Analysis

**Figure 2:**
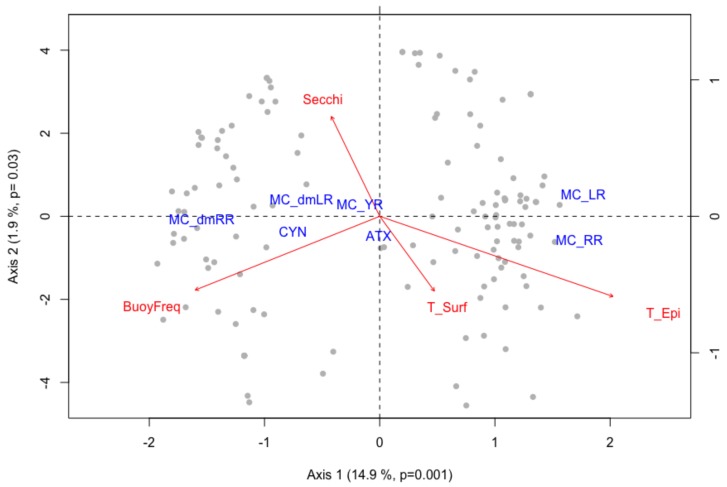
RDA biplot of the toxin quota (toxin μg/chlorophyll-a μg; Hellinger transformed due to many zeros) of the five microcystin variants (MC-YR; MC-dmLR; MC-LR; MC-RR; MC-dmRR), cylindrospermopsin (CYN) and anatoxin (ATX). The vectors represent the environmental variables: epilimnetic temperature (T_Epi), surface temperature (T_Surf) and the log transformed Secchi depth (Secchi) and maximum buoyancy frequency (BuoyFreq). Length and direction of vectors indicate the strength and direction of the relationship.

The ordination analysis showed a clear delineation among toxin variants in the EMLS lakes (Figure 2). Lakes with MC-dmLR and MC-dmRR were clustered on the negative-value side of the canonical axis 1, with MC-dmLR occupying the positive-value side of axis 2 and MC-dmRR the negative side of the second axis. Lakes with MC-LR and MC-RR were grouped on the positive-value side of axis 1, and on the positive- and negative-value side of axis 2, respectively. MC-YR demonstrated the highest positive correlation with the canonical axis 2 (r = 0.24). ATX correlated significantly with the negative-value side of axis 2 while CYN correlated negatively with the canonical axis 1. The permutation test confirmed the significance of the canonical analysis (*p* = 0.001 for axis 1, *p* = 0.03 for axis 2).

The redundancy analysis for toxin concentration and toxin quota data yielded the same results (Table 2). Both the distribution of toxin concentrations and toxin quota were defined by epilimnetic temperature (T_Epi), surface temperature (T_Surf), buoyancy frequency (BuoyFreq) and Secchi depth (Secchi). Therefore, in Figure 2, we present only the biplot for the toxin quota, since the plot for the toxin concentration was almost identical. Marginal tests showed that T_Epi, T_Surf, BuoyFreq and Secchi were all significant in determining the toxin variant ordination (Table 2). T_Epi correlated closely with the negative-value side of the axis 1 (r = −0.62). T_Surf and BuoyFreq had a stronger correlation with the axis 2 (r = −0.55 and r = −0.54). The positive-value side of the second axis correlated strongly with Secchi (r = 0.73). Variance partitioning showed that T_Epi explained 7.3%, T_Surf 2.5% and BuoyFreq 1% of the variance (Figure S1a), while the Venn diagram on T_Epi, T_Surf and Secchi demonstrated 11%, 7% and 1% of variance explained, respectively (Figure S1b). 

### 2.3. Toxin Diversity Index and Environmental Parameters

The environmental parameters, maximum depth (D_Max_), latitude (Latitude), epilimnetic temperature (T_Epi), maximum buoyancy frequency (BuoyFreq) and Secchi depth (Secchi), were selected (stepwise selection) as the best explanatory variables in the final model for both the TDI and Richness. The negative binomial generalized linear model showed a significant positive effect of latitude, and a significant negative effect of maximum buoyancy frequency in defining the TDI on a continental scale (Table 3). In the case of Richness, the model showed again a significant positive effect of latitude and a significant negative effect of maximum buoyancy frequency. Additionally, epilimnetic temperature (T_Epi) had also a significant positive effect, while Secchi depth had a significant negative effect in determining Richness. Both of these factors, however, explained less variance than latitude and buoyancy frequency (X^2^, Table 3).

Each toxin quota was tested separately against the TDI to reveal responses in individual toxins to changes in overall toxin diversity (Table 4). The negative binomial generalized linear model showed that the variants MC-YR and MC-dmLR increased significantly (*p* < 0.05) with increases in TDI. The response of CYN and ATX to increases in the TDI were positive and highly significant (*p* < 0.01). Positive trends in total microcystin, MC-RR and MC-dmRR were also determined but without any statistical significance (*p* > 0.05). MC-LR was the only toxin variant that showed a negative trend (red arrow) to increases in toxin diversity, although lacking statistical significance. Similarly, all toxin quota were tested against Toxin Richness. In this case, all toxin quota increased significantly with toxin richness apart from MC-dmLR and MC-LR that, although showing a positive trend, were not significant (Table 4).

## 3. Discussion

Our study shows that MCs were, by far, the most abundant cyanotoxins across the European lakes in our dataset, being detected at greater frequency than either CYN or ATX (Table 1). However, it is important to note that we analysed only the intracellular toxin content on filter samples, which might have resulted in an underestimation of CYN or ATX concentrations, as they can be largely extracellular [36]. We found that among the microcystins, MC-LR was only the third most abundant microcystin variant, after MC-YR and MC-dmLR (Table 1). MC-dmRR was the least common toxin in the EMLS lakes, but showed the highest concentrations (up to 14 μg/L). Furthermore, CYN was detected less frequently, but, in several cases, it was the only toxin detected that could indicate that CYN producers might have a potential to exclude the producers of other toxin variants (Figure 1). CYN can be present over extended period in aquatic systems, since it can be produced by a succession of different bloom species. For example, in Lake Albano (Italy), a succession in CYN production by *Cylindrospermopsis raciborskii* to *Aphanizomenon ovalisporum* lead to the toxin being present in the system from early summer until early autumn [43]. ATX also occurred as a single toxin, i.e., not in complex mixtures with other cyanotoxins, albeit at lower concentrations (Figure 1). A concrete example is the ongoing substitution of *P. rubescens* (mainly a MC-dmRR producer) by *Tychonema bourrellyi* (ATX-producer) in Lake Garda (Italy). Over the last decade, a shift in dominance between these two species caused an increase in ATX at the expense of MC-dmRR [44]. However, and perhaps more typically, there are studies that highlight ATX dominance during short periods of time only, most likely because MC producing taxa take over after a short period of dominance by ATX producers [45]. These results indicate that risk assessment should be broader and address other toxin variants than just the well-known MC-LR variant. A similar conclusion has been reached by other studies, with relevance for human risk assessment but equally for ecosystem functioning [37]. 

In the cyanobacterial literature, it is entirely customary to discuss data on presence of toxins in direct relation to the cyanobacterial taxa that produce them. However, here, we only present data on toxins, no information on the taxonomic composition of the phytoplankton communities of the EMLS lakes is given. Why is this the case? To begin with, this goes back to one of the key principles underlying the EMLS, one of complete data integration. As explained in the methods’ nutrients, HPLC pigments or toxins come from one instrument, operated by one person. Samples for microscopy were taken, but each participating laboratory counted these locally, using different quality microscopes and varying levels of taxonomic expertise. Given a recent discussion [46] on problems with the trustworthiness, even at the genus level, of a long-term phytoplankton dataset in which all samples were counted by a small team of experts, supervised by the same person over the years, we could not trustfully use microscopic counts from so many different labs in our study. Moreover, in recent studies, there is a strong tendency to focus on key functional traits as the focal point of phytoplankton ecology (e.g., [47]). Cyanobacterial key traits, rather than taxonomic relatedness are also the basis for successful management of cyanobacterial blooms [48]. For the purpose of this study, we examined toxins as functional traits and aimed to study how much we can understand about the spatial drivers of toxin abundance by just focusing on the traits themselves. This trait-centred view may be further supported by the fact that all countries base their assessment of the risks of toxic cyanobacteria for the consumption of drinking water or food directly on toxin concentrations and not on taxonomic information [31]. A further EMLS paper is in preparation where we compare traits-pigments from HPLC and size related traits from flowcytometry-with phylogenetic information (16s rRNA) and functional genes (toxin synthetases) to better understand the occurrence of cyanobacteria at the continental scale.

Functional trait diversity of cyanobacteria explains the coexistence or succession of the different cyanobacteria species under diverse environmental conditions or lake settings [11]. However, species-specific toxin production is rarely attributed to environmental factors with certainty. Clusters of genes encoding different cyanotoxin classes can be selectively present in different cyanobacteria species and strains, but mutations can also turn a toxic genotype into non-toxic, under conditions that are not exhaustively studied [49]. Phylogenetic analysis on the evolutionary age of the MC/NOD synthesis pathways implied that all cyanobacteria are potential MC producers [50]. Reported data from Lake Great Prespa (Greece–Macedonia) showed that, although certain isolated cyanobacteria species produced more specific MC variants, they all had the potential to produce all the analyzed MC variants, just in smaller quantities. Toxin concentrations are the result of (i) species abundances; (ii) the abundance of potentially toxigenic genotypes; (iii) the type of toxins that can be produced by those strains; (iv) the cellular quota of the toxins; and, finally, (v) how all these levels are controlled by environmental settings. In short, the product of toxigenic cyanobacterial biomass x cellular quota determines the toxin concentration in the lake. In terms of environmental drivers, there are those that control cyanobacterial growth and losses (growth – losses = biomass), and most of these are well studied, in particular phytoplankton resources like phosphorus, nitrogen and light [51]. Moreover, cyanobacterial growth is strongly temperature dependent [52]. Population losses are driven by factors like lysis, grazing and parasitism [53]. Many of the factors that determine biomass have also been found to have an effect on toxin quota (e.g., [28,54,55]). 

Indeed, in line with the overlap in environmental control of biomass and quota from the literature, the distribution of toxin concentrations and quota in EMLS were explained by the same set of environmental predictors. Previous field studies showed elevated MC concentrations in lakes with both low or high cell abundances [56]. MC concentration per unit biomass can vary considerably from one bloom to another, or even within the same bloom. On the other hand, stable toxin quota have also been observed [16]. Toxin quota can vary greatly within a toxin producing species, e.g., ranging from 0–5 μg/mg of dry cyanobacteria biomass [57]. From a management point of view, understanding what drives low toxin quota during high cyanobacterial biomass or high toxin quota during low cyanobacterial biomass versus simply looking at the overall toxin concentrations would be helpful to better understand variation in toxin concentrations and the risks they pose for use of the water systems [58]. Even in oligotrophic lakes that typically have low cell densities, the cyanobacterial biomass may accumulate at surface and form scums at leeward shores [59], potentially leading to highly localized toxin concentrations, especially when cells possess considerable toxin quota. In contrast, the influence of environmental factors on strain composition is hardly understood.

The ordination model showed that temperature effects were mostly responsible for the distribution of the different toxins at a continental scale (Table 2). Interestingly, a significant grouping of lakes with MC-LR and MC-RR on the one hand, and lakes with MC-dmLR and MC-dmRR on the other hand was found (Figure 2). According to these results, lakes with high MC-LR contents would be more likely to have the MC-RR variant as well, while lakes with MC-dmLR are likely to also produce MC-dmRR (Figure 2). Epilimnetic temperature accounted for the delineation of lakes with MC-LR and MC-RR, while lakes with MC-dmLR and MC-dmRR were positively associated with increased buoyancy frequency (Figure 2). This division in lake and toxin groupings indicates that lakes characterized by frequent wind mixing (low buoyancy frequency) and elevated temperature support producers of MC-RR and MC-LR [60]. An example of this type of lake and conditions is Lake Taihu (China), dominated by *Microcystis* [4]. Conversely, in deeper and more stable stratified waters, the co-dominance of MC-dmRR and MC-dmLR may be attributed to buoyant species that can accurately regulate their position in the water column providing them a stable position in the metalimnion, like in particular *Planktothrix rubescens* (e.g., [61,62]). See below for more in-depth discussion.

Our results did not indicate that either total phosphorus or total nitrogen concentrations had a significant impact on the distribution of toxin concentrations or toxin quota (Table 2). As discussed earlier, studies have shown that increased nutrients are linked to increased growth rates and toxin production (references within [26] and results of [8]). However, there is also contradictory evidence that, for example, nitrogen availability promoted cell growth, but it did not directly influence toxin production [63]. Hence, there is no consistent evidence supporting a causative relationship between nutrient supported growth and toxin production. In our analyses at the continental scale, nutrients in sharp contrast to temperature effects did not emerge as control factors. We could argue that nutrients would potentially play a role in the occurrence of the individual toxin variants through supporting high cyanobacterial biomasses or toxin quota, but, according to the results from our study, nutrients would not be the predictor that would select among the different toxin variants, or affect toxin diversity.

**Figure 3:**
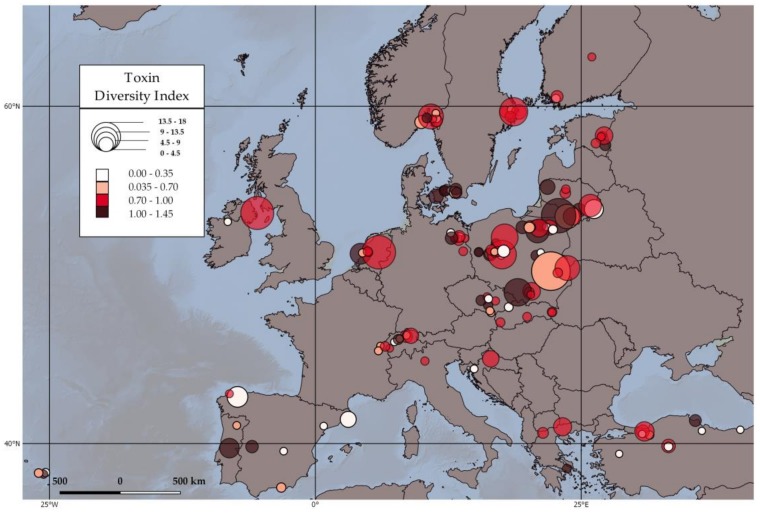
Map of the Toxin Diversity Index (TDI) of the 137 EMLS lakes, calculated using the Shannon equation. TDI is categorized in four classes with higher colour density (red) representing higher toxin diversity and lower colour density (white) lower toxin diversity. The radius of the markers corresponds to the total toxin concentration in μg/L.

Toxin diversity and richness showed a significant increase with latitude, which means that northern areas exhibited a higher toxin diversity (Figure 3, Table 4). In a parallel study based on the same lake dataset, Mantzouki et al. (in preparation) showed that, during summer 2015, a significantly higher algal-and specifically cyanobacterial-biomass was found in the Boreal climatic zone, compared to lakes in Continental and Mediterranean climate. The higher cyanobacterial biomass was potentially explained by the heat wave that occurred mainly in northern European regions (NOAA online data). The majority of the Boreal EMLS lakes (75%) were sampled during a two week-period where temperature anomalies exceeded the local long-term average summer temperature by +5 °C, compared to a much smaller temperature anomaly in the Mediterranean lakes (+1.8 °C). Cyanobacteria growth rate steeply increases with water temperature until about 25 °C, and plateaus at about 28 °C, while temperature can be detrimental when it exceeds 33 °C [64]. In 90% of the Boreal and Continental lakes, with the +5 °C temperature difference, epilimnetic temperatures approached, but did not exceed 25 °C, giving a higher potential to northern European strains to reach their optimum growth rate. Contrastingly, the Southern European strains were already on the plateau of their growth curves and a 2 °C warming did not add any growth potential. Although a +5 °C versus a +2 °C difference may explain why cyanobacteria in the northern Europe, may have caught up with those in the south, it would not explain why cyanobacterial biomass in the north would actually be higher. Clearly, there are differences in temperature responses, both between and within cyanobacterial species [65]. The rather extreme +5 °C temperature anomaly could have altered community composition and/or have favoured cyanobacterial genotypes with an exceptional set of thermal reaction norms [65]. The extremely warm summer of 2015 in the north is likely to have selected genotypes, which are at the extreme upper-warm-end of the “set of reaction norms” that have evolved locally. This could potentially explain why cyanobacteria in the Boreal lakes developed higher biomass.

High buoyancy frequency, as a proxy for water stability, had a significantly negative effect on the TDI and richness of the EMLS lakes (Table 3). In strongly stratified lakes, in particular those with an oligo-mesotrophic state, highly selective conditions arise with a strict spatial separation of light at the surface and nutrients at depth. This leads to the selection of metalimnetic species with a very specific set of functional traits, like well-controlled buoyancy regulation and elevated phycoerythrin content [62]. Under such conditions where a single cyanobacterium monopolizes the resources, we may expect that the low cyanobacterial diversity leads to a low toxin diversity (Table 3). On the other hand, in lakes with less stringent environmental conditions, like the more shallow and eutrophic lakes in the EMLS dataset, the scope for co-existence of several less specialised cyanobacterial species is enhanced, hence a more diverse toxin community might be established. 

In the EMLS dataset, high MC concentrations did not exclude production of the other two toxin classes (neurotoxins, cytotoxins), but rather increased the probability of CYN and ATX occurrence together with MCs, resulting in an increase in the TDI (Table 4). A diverse representation of toxin variants can increase the relative toxic potential of a lake ecosystem [66]. As the different toxin classes have different modes of action and target different organs, separate toxicity assessments are required [34], but ultimately these separate assessments need to be combined to evaluate the overall toxicity risks. The relative toxic potential of the cyanotoxin mixture is calculated as the sum of the relative abundance of each toxin variant multiplied by a defined Toxicity Equivalent Factor based on LD_50_ values, for each toxin class separately (e.g., neurotoxins vs. hepatotoxins), as proposed in [34]. As the presence of different toxin classes increases significantly with toxin diversity (Table 4), the differentiated toxic potential would have ramifications for understanding the overall risks of blooms in a lake with an elevated TDI. A higher toxin diversity would potentially lead to higher stability in overall toxicity within a bloom, since, if one toxin declines, another may increase, leading to persistence in overall toxicity [26]. To make things worse, it may not be sufficient to look at the sum of toxins present (additive effects), since synergistic effects of cyanotoxin mixtures pose a potential risk to humans, animals and aquatic ecosystems [67,68,69]. 

To conclude, we demonstrated that temperature effects were largely responsible for the distribution of the different cyanotoxins on a continental scale. Additionally, we showed that temperature related mechanisms lead to the selective development of well-adapted strains of cyanobacteria that would reduce toxin diversity, potentially promoting dominance by a few highly toxic strains. Further, high buoyancy frequency, as a proxy for water stability, had a significantly negative effect on the TDI and toxin richness. Overall, our study provided the - perhaps surprising - result that at this large-scale temperature rather than classic drivers of toxic blooms like nutrients, determines the distribution of toxins.

## 4. Materials and Methods

### 4.1. Sampling Survey

The European Multi Lake Survey (EMLS) was organized by 26 European countries during summer 2015, with each participating group using their own financial means to conduct their sampling. Since the EMLS was a voluntary effort, individual countries contributed samples from lakes that they routinely sampled, and these were typically lakes with a history of eutrophication. A total of 369 lakes were sampled using standardized protocols for sampling, processing and preserving. Sampling took place during the two warmest weeks of the summer, which was specific for each region. Data collectors identified the correct sampling period using long-term air temperature data spanning at least 10 years. The sampling location was either the historically sampled location, or the centre of the lake if the lake was not routinely sampled. A temperature profile, with a minimum required resolution of 0.5 m sampling intervals, served to define the sampling depth. An integrated water column sample, which will henceforth be referred to as epilimnetic sample, was taken from 0.5 m below the surface until the bottom of the thermocline. This was defined as the point where there was a ≥1 °C temperature change per meter lake depth. If the lake was shallow, then the entire water column was sampled until 0.5 m above the lake bottom. 

All data collectors constructed a simple device using a stoppered hose of the correct length in order to acquire the epilimnetic sample. The hosepipe was lowered with the bottom end open in the water, at a depth of just under the end bottom of the thermocline. When the hosepipe was vertical and the water level was visible at the surface layer of the hosepipe, then the stopper was inserted to create vacuum pressure. The bottom end of the hosepipe was pulled to the surface to collect the epilimnetic sample in a bucket. The diameter of the hosepipe was appropriate to sample the required water volume (about 5–10 L for hypertrophic and eutrophic, 15–30 L for mesotrophic and oligotrophic lakes) for the analyses, in an acceptable number of runs. The first three sampling runs served the purpose of rinsing the hosepipe, the sampling bucket and the plastic rod. The subsequent runs were the water sample taken for analysis. The water sample in the bucket was mixed adequately before being divided into different bottles for further processing prior to analysis. For pigment and toxin analyses, a volume of 50–250 mL for hypertrophic and eutrophic lakes, and 500–1000 mL for mesotrophic to oligotrophic lakes, was filtered through 47 mm Glass fibre filters (GF/C or GF/F or similar) using a filtration device. The filters were stored in −20 °C and in the dark until shipping. For analyses of total nutrients, unfiltered water subsamples of 50 mL were stored in −20 °C until shipping. All samples were shipped frozen using dry ice in Styrofoam boxes.

All participating countries took part in a one-week training school to discuss and practice all field procedures. All samples were shipped to and stored at the University of Wageningen (Netherlands) until further analysis. Each of the nutrients, pigments and toxins analyses were done in one dedicated laboratory, by one operator on one machine, to minimize analytical errors and maximize integration of the datasets. Specifically, the nutrients, MCs and NOD analyses were done at the University of Wageningen; the pigment analysis at the University of Amsterdam; and the CYN and ATX analysis at the German Environment Agency.

### 4.2. Cyanotoxin Analysis

In the laboratory, frozen filters were transferred to 8 mL glass tubes and placed for two hours in a freeze-drier (Alpha 1-2 LD, Martin Christ Gefriertrocknungsanlagen GmbH, Osterode am Harz, Germany). The freeze-dried filters then used for the Liquid Chromatography with tandem Mass Spectrometry detection (LC-MS/MS) analysis of microcystins (MCs), nodularin (NOD), cylindrospermopsin (CYN) and anatoxin (ATX) as they are described below. 

#### 4.2.1. Microcystins (MCs) and Nodularin (NOD) Analysis

For the extraction of MCs and NOD, 2.5 mL of 75% hot methanol −25% ultrapure water (*v*/*v*) was added to the freeze-dried filters, which were then sealed with a screw cap and placed for half an hour at 60 °C. Subsequently, the extract was transferred to a clean 8 mL glass tube. This extraction procedure was performed three times for each filter. The supernatants of the repeated extraction procedure were combined to a final volume of 7.5 mL and then dried in a Speedvac (Thermo Scientific Savant SPD121P, Asheville, NC, USA). After that, the extracts were reconstituted in 900 μL 100% MeOH. The reconstituted samples were transferred into 2 mL Eppendorf vials with a 0.22 μm cellulose-acetate filter and centrifuged for 5 min at 16,000× *g* (VWR Galaxy 16DH, Boxmeer, Netherlands). Filtrates were transferred to amber glass vials for the analysis.

The LC-MS/MS analysis was performed on an Agilent 1200 LC and an Agilent 6410A QQQ (Waldbronn, Germany). The extracts were separated using a 5 μm Agilent Eclipse XDB-C18 (4.6 mm, 150 mm column, Agilent Technologies, Waldbronn, Germany) at 40 °C. The mobile phase consisted of Millipore water (*v*/*v*, eluent A) and acetonitrile (*v*/*v*, eluent B) both containing 0.1% formic acid at a flow rate of 0.5 mL/min with the following gradient program: 0–2 min 30% B, 6–12 min 90% B, with a linear increase of B between 2 and 6 min and a 5 min post run at 30% B (as described in [35]). The injection volume was 10 µL. Identification of the eight MC variants (MC-dmRR, MC-RR, MC-YR, MC-dmLR, MC-LR, MC-LY, MC-LW and MC-LF) and nodularin (NOD) was performed in the positive Multiple Reaction Monitoring (MRM) with the following transitions: MC-dmRR 512.8 *m*/*z* [M + H]^+^ to 135.1 quantifier, MC-RR 519.8 *m*/*z* [M + H]^+^ to 135.1 quantifier, MC-YR 523.3 *m*/*z* [M + H]^+^ to 135.1 quantifier, MC-dmLR 491.3 *m*/*z* [M + H]^+^ to 847.6 quantifier, MC-LR 498.3 *m*/*z* [M + H]^+^ to 135.1 quantifier, MC-LY 868.4 *m*/*z* [M + H]^+^ to 163.0 quantifier, MC-LW 891.5 *m*/*z* [M + H]^+^ to 163.0 quantifier, MC-LF 852.5 *m*/*z* [M + H]^+^ to 163.0 quantifier and NOD 825.5 *m*/*z* [M + H]^+^ to 135.1 quantifier [35]. Mass spectrometric parameters are given in [70]. Each MC variant was quantified against a calibration curve. The calibration curves were made using certified calibration standards obtained from DHI LAB Products (Hørsholm, Denmark). The limit of detection (LOD) for a 250 mL sample was: 0.0489 µg/L for MC-dmRR, 0.0358 µg/L for MC-RR, 0.0050 µg/L for MC-YR, 0.0054 µg/L for MC-dmLR, 0.0086 µg/L for MC-LR, 0.0817 µg/L for MC-LY, 0.0531 µg/L for MC-LW, 0.0206 µg/L for MC-LF and 0.0048 µg/L for NOD. The limit of quantification (LOQ) for a 250 mL sample was: 0.0489 µg/L for MC-dmRR, 0.0358 µg/L for MC-RR, 0.0050 µg/L for MC-YR, 0.0054 µg/L for MC-dmLR, 0.0086 µg/L for MC-LR, 0.0817 µg/L for MC-LY, 0.0531 µg/L for MC-LW, 0.0206 µg/L for MC-LF and 0.0048 µg/L for NOD. 

#### 4.2.2. Cylindrospermopsin (CYN) and Anatoxin (ATX) Analysis

For the extraction of CYN and ATX, 1.5 mL of 0.1% formic acid (FA) was added to the freeze-dried filters, sonicated for 10 min, shaken for 1 hour and then centrifuged. This extraction procedure was repeated two more times and the combined supernatants were dried in a Speedvac (Eppendorf, Germany). Prior to analysis the dried extracts were re-dissolved in 1 mL 0.1% FA and filtered (0.2 µm, PVDF, Whatman, Maidstone, UK). 

LC-MS/MS analysis was carried out on an Agilent 2900 series HPLC system (Agilent Technologies, Waldbronn, Germany) coupled to a API 5500 QTrap mass spectrometer (AB Sciex, Framingham, MA, USA) equipped with a turbo-ion spray interface. The extracts were separated using a 5 mm Atlantis C18 (2.1 mm, 150 mm column, Waters, Eschborn, Germany) at 30 °C. The mobile phase consisted of water (*v*/*v*, eluent A) and methanol (*v*/*v*, eluent A) both containing 0.1% formic acid, and was delivered as a linear gradient from 1% to 25% B within 5 min at a flow rate of 0.25 mL/min. The injection volume was 10 µL. Identification of CYN and ATX was performed in the positive MRM mode with the following transitions: CYN *m*/*z* 416.1 [M + H]^+^ to 194 (quantifier) and 416.1/176, and ATX *m*/*z* 166.1 [M + H]^+^ to 149, 166.1/131, 166.1/91, 166.1/43 (quantifier). Mass spectrometric parameters are given in [71]. Certified reference standards were purchased from National Research Council (Ottawa, ON, Canada). The limit of detection (LOD) for both ATX and CYN was 0.0001 µg/L and the limit of quantification (LOQ) was 0.0004 µg/L for a 250 mL sample.

### 4.3. Nutrient Analysis

Sample bottles were acid washed overnight in 1M HCl and rinsed with demineralized water before usage. Nutrients were measured using a Skalar SAN+ segmented flow analyser (Skalar Analytical BV, Breda, NL) with UV/persulfate digestion integrated in the system. Total phosphorus and nitrogen were measured in unfiltered subsamples, following Dutch standards protocols [72,73]. The limit of detection (LOD) was 0.02 and 0.2 mg/L for total phosphorus and total nitrogen respectively.

### 4.4. Pigment Analysis

The analysis of pigments was modified from the method described by [74]. All filters were freeze dried. Filters (45 mm GF/C and GF/F) were cut in half, placed in separate Eppendorf tubes, and kept on ice until the end of the procedure. We added 600 µL of 90% acetone to each tube along with a small amount of 0.5 mm beads. To release the pigments from the phytoplankton cells, filters were placed on a bead-beater for one minute. Next, they were placed in an ultrasonic bath for ten minutes to increase the extraction yields. This procedure was repeated twice to ensure a complete extraction of the total pigment content from the filters. To achieve binding of the pigments during the High-Performance Liquid Chromatography (HPLC) analysis, 300 µL of a Tributyl Ammonium Acetate (1.5%) and Ammonium Acetate (7.7%) mix were added to each tube. Lastly, samples were centrifuged at 15.000 rpm and 4 °C for ten minutes. 35 µL of the supernatant from both Eppendorf tubes of a filter, were transferred into a HPLC glass vials. Pigments were separated on a Thermo Scientific ODS Hypersil column (250 mm × 3 mm, particle size 5 μm) in a Shimadzu HPLC (Shimadzu corporation, Kyoto, Japan) and using a KONTRON SPD-M2OA diode array detector (Shimadzu corporation, Kyoto, Japan). The different pigments were identified based on their retention time and absorption spectrum and quantified by means of pigment standards.

### 4.5. Response Variables and Environmental Parameters

Our focal response variables were the toxin variants MC-dmRR, MC-RR, MC-YR, MC-dmLR, MC-LR, CYN and ATX. We also calculated the toxin quota as the ratio of each toxin variant concentration (μg/L) and the chlorophyll-a concentration (μg/L). The latter was used as a proxy for the total phytoplankton biomass.

We used the environmental parameters latitude (Latitude), longitude (Longitude), Secchi depth (Secchi), sampling depth (D_Sampl_), maximum depth (D_Max_), total phosphorus (TP), total nitrogen (TN), surface temperature (T_Surf), epilimnetic temperature (T_Epi), maximum buoyancy frequency (BuoyFreq) and light climate (Z_eu_/Z_mix_).

Latitude, longitude, secchi depth, sampling depth and temperature profiles were measured directly in the field at all sites. We interpolated all the temperature profiles at a 0.5 m resolution to standardize the data, as most of the profiles were obtained at a higher resolution than the required minimum interval of 0.5 m. From the interpolated profiles, we calculated the epilimnetic temperature as the average temperature from surface until the bottom of the thermocline. The surface temperature value corresponded to the surface temperature.

We calculated maximum buoyancy frequency (BuoyFreq) as a metric of stratification strength [75]. In the rLakeAnalyzer package [76] in R 3.3.3. statistical software (R Core Team, Vienna, Austria), temperature profiles were used to estimate profiles of buoyancy frequency (N^2^). N^2^ is defined as the Brunt–Väisälä equation: N^2^ = −(g/ρ_0_) × (δ_ρ(z)_/δ_(z)_), where *g* is the gravitational acceleration, ρ_0_ is the density at each depth, and δ_ρ(Z)_/δ_z_ is the density gradient. The rlakeAnalyzer uses temperature profiles (in our case of 0.5 m resolution) to determine the density gradients, applying thermodynamic equations specific to freshwater systems [77]. The maximum value of buoyancy frequency generated from the profile was used as an indication of depth where stratification was the strongest.

The ratio (Z_eu_/Z_mix_) of euphotic depth (Z_eu_) to the mixing depth (Z_mix_) describes the light climate that phytoplankton experience while circulating underwater [78]. We calculated Z_eu_ as Z_eu_ = 2 × Z_SD_ (Secchi depth). As Z_mix_ in shallow lakes, we used the sampling depth when N^2^ was 0, or the top of the metalimnetic depth when stratification was present. In deep lakes, we used the top of the metalimnetic depth. This was calculated as the depth where the steepest density gradient was found [76].

We used a total of 137 out of the 369 sampled lakes in order to test our hypotheses. Selection of a smaller subset was justified since, in some samples, several environmental variables were missing either due to shipping issues or due to deviations from preservation protocols that could affect the integrity of the dataset. Additionally, in order to build the ordination approaches described further down, lakes that had zero concentrations in all toxin variants needed to be excluded to build a meaningful similarity matrix of toxins. Hence, it was necessary to sacrifice a big number of lakes to build a concise dataset where all parameters could be used to select the right model and test the toxin distribution in the EMLS. Samples that were below the limit of detection (LOD), i.e., a toxin signal was detected qualitatively, but it was too weak to quantify, and was assigned a very small value of half the limit of quantification (LOQ), enabling their inclusion in the analysis. The toxins MC-LF and NOD—which were found only in two lakes—and MC-LY and MC-LW—which were absent from all lakes—were removed from the analysis following the approach of [79] for the most rare species in a dataset. Any statistical results included in this paper correspond to the subset of the 137 EMLS lakes. Supplementary Material provides a table with information on the total number of lakes where toxin variants were (a) not present (no toxin signal); (b) present; and (c) missing, for the 369 EMLS lakes (Table S1). All response variables and environmental parameters, along with the Toxin Diversity Index (TDI) and toxin Richness of the 137-lake subset are provided in the Supplementary Material (Table S2).

### 4.6. Statistical Analysis

The geographical distribution of the toxin variants (Figure 1a) and their toxin quota (Figure 1b) were mapped with QGIS 2.18 Las Palmas (QGIS Development Team, Girona, Spain). We use pie charts to show the percentage of each toxin variant in each sampled lake. 

To investigate the relationship between the toxin concentration/quota, distribution and the environmental parameters, we used canonical redundancy analysis of principal coordinates (RDA) with permutation test (9999 permutations). Analysis of variation inflation factor (VIF) allowed us to use all sampled environmental variables (as mentioned in Section 4.5) to test the relationship between toxin concentrations/quota and environmental variables. Most of the environmental parameters were standardized using log_10_ transformation (except for surface and epilimnetic temperature). The toxin concentration and toxin quota matrices were standardized by Hellinger transformation [80]. A stepwise elimination of environmental predictors was applied to find the set of parameters that could best explain the ordination of the toxin concentrations/quota. The selected environmental parameters were: surface temperature (T_Surf), epilimnetic temperature (T_Epi), maximum buoyancy frequency (BuoyFreq) and Secchi depth (Secchi). Significance of the ordination was provided performing ANOVA analysis. 

We used the Shannon–Wiener index to calculate a Toxin Diversity Index (TDI) based on the EMLS toxin quota of 137 lakes. The number of toxins (“Richness”) per lake was also calculated based on the same data. A negative binomial generalized linear model was used to determine the effect of significant environmental parameters on defining the TDI. The same model was used to determine the relation of each toxin variant to TDI.

For all the above analyses, we selected the most significant environmental parameters using stepwise selection, based on the Akaike Information Criterion. All statistical analyses were performed in R 3.3.3 [81] using mainly the vegan [82] and MASS [83] packages.

## Figures and Tables

**Table 1:** Summary of toxin variants (Total microcystin: MC-Tot, microcystin YR: MC-YR, microcystin dmLR: MC-dmLR, microcystin LR: MC-LR, microcystin RR: MC-RR, anatoxin: ATX, cylindrospermopsin: CYN, microcystin dmRR: MC-dmRR) ordered by decreasing number of presence in the investigated 137 EMLS lakes.

Toxin Variant	Present (*n* Lakes)	Concentration Range (μg/L)	Limit of Quantification ^1^ (μg/L)	Mean	Stdv
MC-Tot	127	0–17.18		1.20	2.70
MC-YR	113	0–4.92	0.0050	0.14	0.56
MC-dmLR	108	0–3.16	0.0054	0.15	0.50
MC-LR	93	0–3.97	0.0086	0.20	0.55
MC-RR	67	0–3.31	0.0358	0.20	0.50
ATX	54	0–1.33	0.0004	0.03	0.12
CYN	53	0–2.01	0.0004	0.05	0.20
MC-dmRR	52	0–14.89	0.0489	0.52	1.83

^1^ limit of quantification (LOQ) of the LC-MS/MS method measured for an average filtered volume = 250 mL.

**Table 2:** Redundancy analysis showing results of marginal tests for toxin concentrations followed by toxin quota (both Hellinger transformed) based on F-model and 9999 permutations. Epilimnetic temperature (T_Epi), surface temperature (T_Surf), maximum buoyancy frequency (BuoyFreq) and Secchi depth (Secchi) were the predictors that were selected (stepwise elimination) for the constrained analysis. The Adjusted R^2^ (AdjR^2^) estimates the relative quality of the two models. Statistically significant effects are shown in bold.

RDA	AdjR^2^	Predictor	Variance	F	*p*
Toxin Concentrations	0.14	T_Epi	0.05	13.22	**0.001**
		T_Surf	0.02	4.93	**0.002**
		BuoyFreq	0.01	3.17	**0.01**
		Secchi	0.01	2.87	**0.01**
Toxin Quota	0.14	T_Epi	0.05	13.22	**0.001**
		T_Surf	0.02	4.93	**0.003**
		BuoyFreq	0.01	3.17	**0.02**
		Secchi	0.01	2.87	**0.02**

**Table 3:** Summary of the Generalized Linear Model for the Toxin Diversity Index (TDI) and Toxin Richness of toxin quota. Stepwise elimination selected for final model with predictors maximum depth (D_Max_), latitude (Latitude), epilimnetic temperature (T_Epi), maximum buoyancy frequency (BuoyFreq) and Secchi depth (Secchi). Statistical significant variables are shown in bold.

Index	GLM, Family = Negative Binomial	Predictor	Χ^2^	*p*
TDI_quota_	−1.93 + 0.003 D_Max_ + 0.03 Latitude ** + 0.03 T_Epi −24.2 BuoyFreq * − 0.06 Secchi	Latitude	1.21	**0.004**
		BuoyFreq	0.75	**0.02**
		D_Max_	0.08	0.8
		T_Epi	0.24	0.2
		Secchi	0.30	0.15
Richness_quota_	−0.16 + 0.002 D_Max_ + 0.02 Latitude ** + 0.04 T_Epi * −26.3 BuoyFreq *** − 0.09 Secchi **	Latitude	1.49	**0.006**
		BuoyFreq	2.14	**0.001**
		D_Max_	0.41	0.15
		T_Epi	1.13	**0.02**
		Secchi	1.40	**0.007**

**Table 4:** Statistical results of the negative binomial generalized linear model, showing the response of the toxin quota (MC-YR, MC-dmLR, MC-LR, MC-RR, MC-dmRR, ATX and CYN over chlorophyll-a) to increases in Toxin Diversity Index (TDI) and Toxin Richness. Black upward arrows correspond to increases of the toxin variant to increases in the TDI and Toxin Richness, red downward arrows correspond to decreases of the toxin variant when TDI increases. Statistically significant effects are shown in bold (*p* < 0.05).

Toxin Quota	Response When TDI ↑	Χ^2^	*p*	Response When Richness ↑	Χ^2^	*p*
MC-YR	↑	0.10	**0.02**	↑	0.10	**0.01**
MC-dmLR	↑	0.10	**0.02**	↑	0.06	0.06
MC-LR	↓	0.09	0.54	↑	0.3	0.2
MC-RR	↑	0.44	**0.06**	↑ **	0.90	**0.003**
ATX	↑ ***	0.15	**0.0002**	↑ ***	0.19	**<0.0001**
CYN	↑ **	0.38	**0.007**	↑ ***	0.56	**0.0009**
MC-dmRR	↑	0.2	0.85	↑	0.41	**0.02**

highly significant results are marked with “**” for *p* < 0.01 and “***” for *p* < 0.001.

## Supplementary Materials

The following are available online at http://www.mdpi.com/2072-6651/10/4/156/s1, Table S1: Number of lakes where toxin variants (a) were not present (no toxin signal), (b) present, (c) missing values in the entire EMLS dataset (n = 369 lakes), Table S2: Basic information on the study lakes; their physical data, analysed nutrients, pigments, toxins, and calculated indices.
